# Cost-utility analysis of genetic screening in families of patients with germline *MUTYH *mutations

**DOI:** 10.1186/1471-2350-8-42

**Published:** 2007-07-02

**Authors:** Maartje Nielsen, Frederik J Hes, Hans FA Vasen, Wilbert B van den Hout

**Affiliations:** 1 Center for Human and Clinical Genetics, Leiden University Medical Center, the Netherlands; 2 Department of Gastroenterology, Leiden University Medical Center, the Netherlands; 3 The Netherlands Foundation for the Detection of Hereditary Tumours, Leiden, the Netherlands; 4 Department of Medical Decision Making, Leiden University Medical Center, Leiden, the Netherlands

## Abstract

**Background:**

MUTYH associated polyposis (MAP) is an autosomal recessive inherited disorder. Carriers of bi-allelic *MUTYH *germline mutations have a risk of approximately 60% to develop colorectal carcinoma (CRC). In the general population about 1.5% is a heterozygous *MUTYH *mutation carrier. Children of MAP patients have an increased risk of inheriting two *MUTYH *mutations compared to the general population, implicating an increased risk for developing CRC.

**Methods:**

Using data from the literature and Dutch MAP patients (n = 40), we constructed a Markov model to perform a societal cost-utility analysis of genetic screening in MAP families. Genetic screening was done by testing the spouse first and, in case of a heterozygous spouse, also testing of the children.

**Results:**

The cost of genetic screening of families of MAP patients, when compared to no genetic screening, was estimated at €25,000 per quality-adjusted life year (QALY). The presence of Fecal Occult Blood testing (FOBT) population screening only slightly increased this cost-utility ratio to €25,500 per QALY. For a MUTYH heterozygote index-patient, the ratio was €51,500 per QALY. The results of our analysis were sensitive to several of the parameters in the model, including the cost assumed for molecular genetic testing.

**Conclusion:**

The costs per QALY of genetic screening in families of MAP patients are acceptable according to international standards. Therefore, genetic testing of spouses and/or children should be discussed with and offered to counselees.

## Background

MUTYH-associated polyposis (MAP), reported in 2002 by Al Tassan et al, is the first autosomal *recessive *inherited disorder known to result in an increased risk for developing colorectal adenomas and carcinoma [1]. Bi-allelic carriers (with mutations in both alleles of the MUTYH gene, i.e. a MAP patient) develop polyposis and subsequently colorectal carcinoma (CRC) in the majority of cases. Bi-allelic *MUTYH *mutations are found in 10–25% of patients with between 10 and a few hundred adenomas and in 1% of patients with a colorectal carcinoma [2-4]. Patients with more than 10 adenomas are currently being offered *MUTYH *mutation analysis. Siblings of a MAP patient have a 25% risk of also having inherited bi-allelic mutations and are eligible for genetic testing.

In contrast, the earlier identified familial adenomatous polyposis (FAP) syndrome, caused by germline mutations in the APC gene, is an autosomal *dominant *inherited disease. Carriers of one mutated *APC *allele develop adenomas and/or CRC and their children have a 50% chance of inheriting the disease. In these families, genetic testing is being offered to children and other family members because of the high probability of inheriting the disease. In a cost comparison, it was shown that predictive genetic testing in FAP kindreds costs less than conventional clinical screening of asymptomatic family members [5].

**Figure 1:**
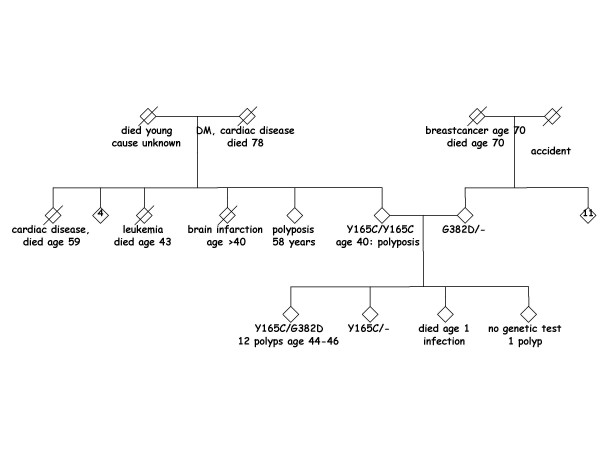
**Family pedigree showing pseudo-dominant inheritance of MUTYH mutation(s)**. Note: '4' and '11' indicate the number of healthy sibs.

Currently, there is discussion about testing spouses and children of MAP patients for *MUTYH *mutations, since spouses have a population risk of approximately 1–2% to carry one (heterozygous) *MUTYH *mutation [1,6]. Consequently, children of the affected index-patient have an increased risk (0.5–1%) of inheriting two *MUTYH *mutations compared to the general population (0.0025–0.01%). An example of such a family is shown in figure 1. The purpose of our study was to explore the economic implications of testing the spouses for carriership and, if the spouse is heterozygous, also their children. Bi-allelic MUTYH children can be screened effectively using colonoscopies. An essential consideration involves the possible implementation of population-wide screening. In the near future, such a screening using Fecal Occult Blood Testing (FOBT) from age 50 years could start in the Netherlands and other European countries. In some countries, including Germany, Austria and Japan, population-wide FOBT-screening is already being implemented on a national or regional scale [7,8]. In the US, adults aged 50 years or older are offered screening by means of FOBT, sigmoidosopy or colonoscopy [9]. Because some of the gain from genetic screening can also be obtained with FOBT screening, we included FOBT screening as a setting in our model.

We will present a cost-utility analysis from a societal perspective estimating the effect on costs and quality-adjusted life expectancy (QALY's) of introducing genetic testing of spouses and, if a mutation is found, the children. We made separate analyses for: (1) the presence or absence of population-wide FOBT screening, and (2) whether the index patient carries one or two *MUTYH *mutations.

## Methods

In deciding whether to instigate genetic screening in MAP families, the balance between societal costs and expected health benefits should be considered. We present our evaluation results in the terms of "additional cost per QALY", making this a cost-utility analysis (CUA). The model estimates effectiveness and cost per child. In our model genetic screening is defined as genetic testing of the spouse and, if the spouse is heterozygous, also testing of the children. In one child families the child is tested without testing the spouse first. The base case analysis is the comparison between two strategies: genetic screening versus no genetic screening in the setting of no FOBT population screening and for a proband with bi-allelic MUTYH mutations. These strategies are also compared in two different settings: 1) presence of FOBT population screening (between ages 50–75) and 2) for a heterozygous MUTYH proband.

**Figure 2:**
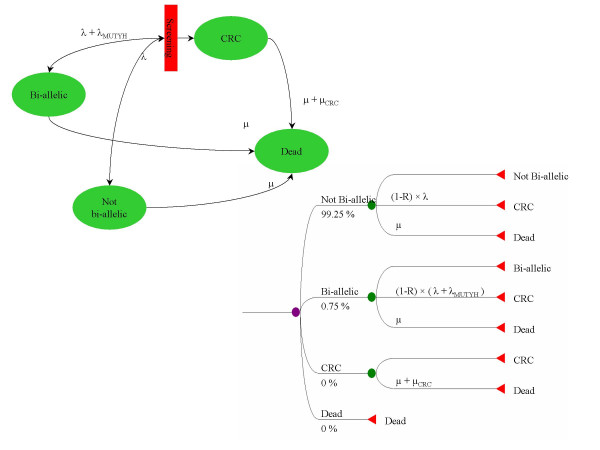
**Model of CRC screening, in flow-diagram en decision-tree representation**. The model distinguishes bi-allelic, non-bi-allelic, and colorectal cancer (CRC) patients. Bi-allelic patients have an increased age-dependent CRC rate compared to non-bi-allelic patients. Moreover, CRC patients have a higher mortality rate than non-CRC patients. Screening blocks CRC incidence. Parameters: μ = General mortality rate, μ_CRC _= Excess CRC-mortality rate, λ = Unrelated CRC-incidence, λ_MUTYH _= MUTYH-related CRC-incidence, R = reduction in CRC incidence due to screening (90% for colonoscopy and 11% for FOBT screening)".

Different screening strategies were compared using a four-state Markov model (figure 2). The model distinguishes bi-allelic, non-bi-allelic (no or one MUTYH mutation), and colorectal cancer (CRC) patients. Bi-allelic patients have an increased, age-dependent CRC rate compared to non-bi-allelic patients. Our model does not include a detailed CRC growth and detection model because of unavailability of data; instead, screening was modelled by reducing the incidence of both MUTYH-related and unrelated CRC (with rate λ and λ_mutyh_, respectively) depending on the type of screening. The baseline mortality rate is depicted as μ and the excess CRC mortality rate as μ_CRC_.

For a full list of assumptions for our model see table 1. Several assumptions for the base case model were analyzed using univariate sensitivity analyses.

**Table 1:** Cost-effectiveness model: input variables and sources

**Variable**	**Base case Assumption **(costs indexed at 2006)	**Range**	**Source**
Prevalence of MUTYH mutations in population	1.5%	1–2%	Al Tassan 2002, Croituro 2004, own data (unpublished) [1;6]
Prevalence of CRC in MAP patients	60%	40–70%	Nielsen 2005, Sampson 2003, Gismondi 2004 [2–4]
Mean age of diagnosis of CRC in MAP patients	49 year		Nielsen 2005, Sampson 2003, Gismondi 2004 [2–4]
Baseline cure rate in MAP patients after CRC	52%	30–60%	*IKA*/Association of Comprehensive Cancer Centers, [13]
Mean number of children per family	2 children		40 Dutch MAP patients (own data, unpublished)
Life expectancy after CRC	Table*		*IKA*/Association of Comprehensive Cancer Centers, [13]
Life expectancy total population	Table*		Central Bureau of Statistics (*Statistics Netherlands*), [32]
Utility after CRC	0.90	0.85–0.95	[24]- utility 9 month after surgery- mean [22] [23]
Spouses compliance to testing	75%	0–100%	See methods, own data, Rowley 2004 [11]
Childrens' compliance to testing	75%	50–100%	Rowley 2004 [11]
Age start screening children	25 year	20–25 year	See text
CRC costs		50–200%	van den Brink 2004 [24]
• initial treatment and recurrence	21,330		
• continuing care	2130/year		
• non-health care (productivity)	11,060		
Costs of genetic testing in a DNA diagnostic laboratory social costs (travel/production)	645€ 95€	200–1000€	Costs genetic testing Leiden University Medical Center (LUMC) [25]
Colonoscopy costs (including polypectomy and complications)	561€ (480€ + 81€)		LUMC, Heitman 2005 [18]
Social costs (travel/production)	70€		Oostenbrink 2004 [25]
Effectiveness colonoscopy (reduction in CRC cases)	90%	70–80%	Winawer 1993 [16]
Effectiveness FOBT (reduction in mortality)	11%	10–30%	Kronborg 2004 [19] Faivre 2004 [20]
FOBT	65€ (18€ medical & 47€ time)		Gyrd-Hansen 1998, Oostenbrink 2004 [7] [25]
Discount rate	4%	3–5%	Oostenbrink 2004 [25]

* Extensive tables, please contact corresponding author for detailed information.

### Families

Families were presumed to be non-consanguineous. In the sensitivity analysis, the number of heterozygotes was varied between 1–2%, according to the difference in frequencies reported in the international literature [4,1,10,6] The mean number of children assumed in the model was based on 34 Dutch MAP pedigrees with at least one child (mean number of children 2.4, median 2).

### Genetic testing

Genetic testing is performed using sequence analysis of the coding regions of the MUTYH gene (see Nielsen [2] et al. for details). All the pathogenic mutations reported in the literature to date can be identified with the methods used. In families with more than one child, testing the spouse, rather than all the children, reduces the required number of tests from one per child to slightly more than one per family. Individual genetic counseling for the spouse is not required, since in most cases he or she can visit the clinic with the index-patient. If the spouse of an index-patient appears to be a *MUTYH *mutation carrier, the children become eligible for separate genetic counseling and genetic testing.

The participation rate of the children, which influences the total costs per child when the spouse is tested first, was assumed to be 75%. The actual compliance rate of children for genetic testing, when the spouse tests positive for a *MUTYH *mutation, is not known. Rowley [11] reviewed the participation rates for genetic testing in hereditary non-polyposis colorectal carcinoma (HNPCC) family members and found a range between 43–92%. The participation rate for genetic testing of children in our study was therefore varied between 50–100%. The participation rate of spouses was also set at 75%, based on our own data, as most spouses (in the Netherlands) opt for genetic testing. In the sensitivity analysis the participation rate of the spouses was varied between 0%, when the children are tested without testing the spouse first in all cases, and 100%.

### MUTYH-related and unrelated CRC

The percentage of MAP patients reported to develop CRC lies between 50–70% of cases [2,4,10]. The age of diagnosis of CRC in MAP patients was based on the age distribution among 73 MAP patients with CRC, as previously published [2]. The phenotypical expression of the disease is not yet fully established. Until now, 7,949 healthy subjects have been tested for *MUTYH *mutations [12] and although no bi-allelic mutation carriers were found, there is still a possibility that some bi-allelic *MUTYH *mutation carriers will *not *develop polyps and carcinoma. Hence, the penetrance of CRC in MAP patients was varied between 40–70% in the sensitivity analysis.

In 2003, the cumulative risk for 0–79 years for CRC in the general Dutch population was 6% (Association of Comprehensive Cancer Centres, ACCC). For the survival of CRC patients we used data from the ACCC [13]. These data were modelled using a so-called cure model [14] in which 52% of patients is cured without excess mortality and the remaining 48% have a constant excess mortality rate of 0.49 per year.

The natural history of CRC in MAP patients may differ from that in the general population. It is possible that MUTYH-related CRC patients may have a better survival than sporadic CRC cases, as reported previously in HNPCC patients [15], or worse survival. Therefore, the 52% baseline cure rate in MAP patients after CRC diagnosis was varied in the sensitivity analysis between 0.30 and 0.60.

### Colorectal colonic screening

Winawer et al. reported that the incidence of CRC was reduced by 90% in patients that had one or more, large (> 1 cm) adenomas removed when periodic colonoscopy and polypectomy were performed [16]. MAP patients have between 10–500 adenomas at a mean age of 50 years, and are apparently not comparable to the standard symptomatic patient (e.g. with rectal blood loss) in whom usually less than ten adenomas are detected at colonoscopy. However, the number of polyps in the MAP patients accumulates over time and, if screening is started from young adulthood, the number of adenomas encountered every two years is expected to be considerably lower. Besides our base case assumption of a 90% reduction, we also included a 70% and 80% reduction in CRC in the sensitivity analysis. The optimal interval between colonoscopies for those at highest risk seems to be two years [17]. Two-yearly colonic screening in our model started at age 25 and ended at 79 years. At the present time, MAP patients are advised to have screening from age 25 years [2], in a sensitivity analysis we set the start of colonic screening also at age 20. The effect of colonic screening was modeled by uniformly reducing the incidence of MUTYH-related and unrelated CRC, resulting in an equal reduction in mortality. Except in the costs, the consequence of changing the interval between colonoscopies was not explicitly incorporated in the model.

The risk of bleeding and perforation per colonoscopy was assumed to be 0.5–0.24%, respectively, based on a recent article by Heitman et al. Mortality after a perforation was reported to be 4.7% [18].

### Population-wide screening

To incorporate population-wide screening in our model, we used the most recently published figures from a large prospective study in Denmark, 'the Funen study', in which biennial screening with (unhydrated) FOBT was used. A significant reduction in mortality of 11% in the screened group was reported after a study period of 17 years, including nine rounds of FOBT. This figure increased to 12% in persons participating in all nine rounds. The better survival rate is the result of detecting the CRC earlier; there was no reduction in the total number of colorectal cancers [19]. Others have found a 16% mortality reduction over an 11 year period, using also (unhydrated) FOBT [20]. Also population-wide screening could well become more effective in the future because of refinements in FOBT and/or other screening techniques [21]. Besides the base case 11% reduction for population-wide screening, we therefore included a 20% mortality reduction in the sensitivity analysis.

### Quality-adjusted life years

Quality-adjusted life years (QALYs) are often used as an effectiveness measure for health-economic evaluations, because they capture both length and quality of life, and can therefore be used to compare a wide variety of diseases and treatments. To adjust for the quality of life, a utility factor is used on a scale from one (perfect health) to zero (as bad as death). In the case of CRC, quality of life can be impaired because of physical discomfort after surgery or the need to carry a stoma, for example. Utility factors reported in the literature vary between 0.83 [22] and 0.981 [23] due to differences in measuring instruments and the length of follow up. In our base case analysis we used a utility factor of 0.90 after incidence of CRC, based on a report by Van den Brink et al [24]. In the sensitivity analysis the utility factor was varied between 0.85 and 0.95.

### Costs

We analyzed the costs from a societal perspective, so besides medical costs, the model also included health-related non-medical costs, including loss of productivity and patients' time and travel costs associated with health care. Only MUTYH- and CRC-related costs were included in the model. Costs were expressed in euros, indexed to 2006 prices.

The costs for genetic testing of the *MUTYH *gene are low compared to other genes, because the gene is relatively small (11.4 kb) and the analysis is not complicated. The standard charge for molecular genetic testing at our DNA laboratory in the LUMC in Leiden is €645. This includes the costs for the actual DNA test, labor time and administrative costs. We added patients' time and travel costs of €95 [25], leading to our base case overall cost estimate of €740 per genetic test. This was varied between €200 and €1000 in the sensitivity analysis. The charge for genetic counseling of children of MAP patients was set at €144, which is the rate for testing family members at our clinic.

Costs associated with CRC were based on a large Dutch study for evaluating the cost-utility of pre-operative radiotherapy in rectal cancer patients undergoing surgery [24], which reported mean total costs of €110,100 (indexed at 2002) per patient. Based on these data we set costs at €21,330 for initial treatment and €2,130 per year for continuing care (indexed at 2006). The costs of loss of productivity after CRC diagnosis were estimated at €11,060 per CRC diagnosis, based on the friction costs method [26]. We used these costs from rectal cancer patients because they were comprehensive and presented in a way that facilitated the use in our model structure. Other publications reported only total costs [27], from which it would not be easy to derive initial costs and annual costs. Moreover, the cost data we used were not inconsistent with the widely variable data reported for CRC patients in general. Because of possibly different costs for colorectal than for rectal cancer patients and the large differences reported in the international literature [18] we varied the total costs per CRC diagnosis by 50–200% in the sensitivity analysis.

Costs for colonoscopy at our clinic are €474 and time and travel were estimated at €70 [25] (indexed at 2006). The costs per bleeding and perforation were assumed to be €3,067 and €29,982, respectively [28].

The costs for population-wide FOBT screening were obtained from the cost-effectiveness analysis by Gyrd-Hansen et al in 1998, based on the outcomes of the "Funen trial" [29]. In this study different strategies were postulated, we used the data from strategy C in table 1 because in this strategy screening starts from age 55 years and is repeated every 2 years, which is most similar to that advised by the EU council (screening between ages 50–75) [30,31]. Their estimated cost-effectiveness of €4,400 per life-year-saved was reconstructed in our own model by assuming medical costs at €18 per individual FOBT. In addition, time and travel costs were assumed to be €47 [25].

### Miscellaneous

Based on data from the Dutch national statistic bureau in 2005, the life expectancy of the general population at birth is set at 77.0 and 81.5 years for men and women, respectively [32]. The Markov model used a life-long time horizon, divided into one-year cycle times and truncated at age 100. Future cost and quality-adjusted life years were discounted at 4%, as advised in Dutch guidelines for economic health care evaluations [25]. We varied the discount rate between 3–5% in our sensitivity analysis. For a full list of assumptions for our model see table 1.

## Results

Table 2 shows the results of the base case analyses. The expected health gain per bi-allelic child was estimated at 2.4 quality-adjusted life years, which is seven quality-adjusted days per screened child. With costs amounting to €470 per screened child, the estimated cost-utility ratio is €25,000 per QALY. In the presence of FOBT screening, the incremental cost of genetic screening -in persons that also will be invited in population screening- increases only slightly to €25,500 per QALY. If the index patient is a heterozygote, the costs per QALY are €51,500 (and €52,500 if population screening is introduced, results not shown). See table 2 for the estimated costs per life year (LY) gained.

**Table 2:** Results of cost-effectiveness (CE) analyses: incremental effect of genetic screening of MAP families compared to no genetic screening, in different settings:

	Base case	With FOBT*	Heterozygote MUTYH indexpatient
Results per child			
- Additional costs	€470	€470	€469
- QALY^# ^gain, discounted	0.018 years (7 days)	0.017 years (6 days)	0.009 years (3 days)
Results per bi-allelic child			
- Additional costs	€58,500	€59,000	€120,500
- LY^† ^gain, undiscounted	6.9 years	6.7 years	6.9 years
- LY gain, discounted	1.4 years	1.4 years	1.4 years
- QALY gain, discounted	2.4 years	2.3 years	2.4 years
Cost effectiveness (in €/LY undiscounted)	8,500	9,000	17,500
Cost effectiveness (in €/LY discounted)	42,000	42,000	86,500
Cost effectiveness (in €/QALY discounted)	25,000	25,500	51,500

*FOBT: Population screening using Fecal Occult Blood Testing, ^†^LY: Life years, ^#^QALY: Quality-Adjusted Life Year

### Sensitivity analyses

**Figure 3:**
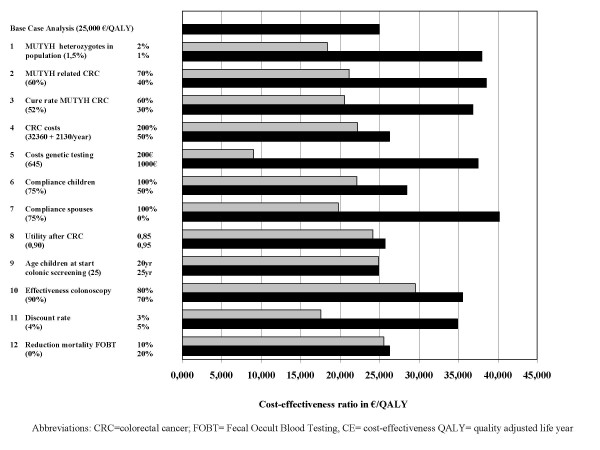
Sensitivity analyses.

The sensitivity analyses (figure 3) showed a considerable variation in possible costs per QALY. The factors dominating the cost-effectiveness analysis were the percentage of heterozygotes (€18,500–38,000 per QALY), percentage of MAP patients that develop CRC (€21,000–38,500 per QALY), mortality due to MAP-related CRC (€20,500–37,000 per QALY), cost of genetic testing (€9,000–37,500 per QALY), compliance of spouses (€20,000–40,000 per QALY), effectiveness of colonoscopy (€29,500–35,500 per QALY) and discount rate (€17,500–35,000 per QALY). Varying the CRC costs, compliance rate of children, utility rate, starting age for screening, and the effectiveness of population-wide screening influenced the cost-effectiveness ratio by less than €4000 per QALY.

## Discussion

A cost-utility analysis, relating costs to the gain in QALYs, is generally accepted as an valuable tool for medical decision-making [33,34]. Since economic considerations are never the only decision criterion, it is impossible to set a strict threshold for the acceptable costs per QALY. A rule of thumb, which is often quoted in the literature, is that costs up to US $20,000 per QALY are certainly acceptable, up to US $50,000 per QALY are probably acceptable, and up to US $100,000 might be acceptable [35,36]. Others have advocated higher thresholds of up to US $200,000 per QALY [37]. Threshold values in terms of Euros would be somewhat higher, since dollars are more valuable than euros (purchasing power parity US $1 = €1.12, [38], June 2006). The Dutch have recently proposed a threshold of €80,000 per QALY [39].

Since the discovery of the MUTYH gene in 2002, hundreds of patients have been diagnosed with MUTYH-associated polyposis. The outcome of our cost-utility analysis shows that genetic testing in MAP families has an acceptable costs per QALY ratio, also after FOBT population-wide screening is introduced (€25,000 and 25,500 per QALY, respectively). The benefit in terms of discounted QALYs per child is seven days per child. This might seem insignificant, but for a child with bi-allelic MUTYH mutations this implies a survival benefit of 2.4 quality-adjusted life years and 6.9 undiscounted life years (table 2). Testing the family of MUTYH heterozygotes, with an estimated cost-utiltiy ratio of €51,500 per QALY, might also be acceptable according to national and international standards, which opens the possibility of cascade screening.

The major limitation of our model is the availability of representative and reliable data. Using univariate (one-way) sensitivity analyses, the consequences of varying different major inputs of the model were investigated. If a spouse does not participate in testing and the children are tested first, the costs increase to €40,000 per QALY. All other key variables led to cost-utility ratios that were more favorable than €40,000 per QALY.

Apart from the availability of reliable data our study has a number of further limitations. First, we did not include the occurrence of duodenal adenomas and duodenal cancer which has been reported in a few MAP cases [40] and which is likely to improve the cost-effectiveness of genetic testing in MAP families. At present, there is not enough information to include this in our cost-effectiveness analysis. MAP patients are currently advised to undergo upper gastro-intestinal tract screening once every 1–5 years, depending on the findings of the previous endoscopy. Second, patients carrying a heterozygous MUTYH mutation might also have a slightly elevated risk for developing CRC. Jenkins et al (2006) found a three-fold relative risk for colorectal cancer in MUTYH heterozygotes in a population-wide case-family study [41]. In contrast a recent meta-analysis of case controls studies showed a non-significant RR of 1,3 (O,99–1,55 for heterozygote MUTYH carriers [12].

Colonoscopic screening of MUTYH heterozygotes from the age of 50 is probably cost-effective, as this was also the case for the general population [27]. Third, although genetic testing is evidently more efficient in larger families, we did not stratify the cost per QALY for the actual number of children in a pedigree. In our opinion, once such genetic testing has been established as a standard procedure, it would be ethically unacceptable in clinical practice to withhold it from smaller families; especially since the participation and number of children only influences the costs per child, not the estimated effectiveness per child who tests positive. Fourth, we did not include the psychosocial aspects of genetic counseling in our analysis. People may experience changes in their functional, emotional or social status after learning their genetic predisposition. These responses, before and after genetic testing, have been characterized in patients at risk for Huntington's disease and inheritable breast cancer [42,43]. Potential adverse effects depend on the severity of the disease in question. We consider these effects to be less for MAP than Huntington or inheritable breast cancer, because the existence of a pre-cancerous stage (adenomas) makes it possible to detect and prevent colorectal cancer by regular colonic screening. Griffith et al. stated that a cost-utility analysis is not suitable to account for the impact of genetic services on the individual, the family and society because of difficulties in measuring non-health benefits. There is a need for further research on the psychosocial impact of genetic services within a health-economics context [44]. For now, we recommend that counselors should consider the psychosocial implications for anyone tested for MUTYH mutations as much as in any other genetic counseling procedure. Fifth, our model is inadequate to evaluate the cost-effectiveness of FOBT screening. For that purpose, more sophisticated models are required and available [29,45]. The data requirements of these models prevent their application to the evaluation of genetic screening. We did included FOBT screening as a setting in our model, because some of the gain from genetic screening can also be obtained with FOBT screening. Therefore, the presence of FOBT screening reduced the gain from genetic screening, but only to a very limited extent. And finally, we did not consider alternative population-wide screening techniques such as colonoscopy and sigmoidoscopy. These screening endoscopies are respectively offered once per 10 and 5 years, in places where these have been introduced, which is probably not frequent enough for MAP patients because they develop tumors in shorter periods.

## Conclusion

Despite several limitations, our model shows that the costs per QALY of genetic screening in families of MAP patients are acceptable according to international standards and we therefore recommend that genetic screening should be discussed with and offered to MAP families in clinical genetic practice.

## Competing interests

The author(s) declare that they have no competing interests.

## Authors' contributions

**MN **collected clinical and all other (cost en epidemiological) data and drafted the manuscript.

**FH **participated in study design and helped drafting the manuscript and critical revision of the manuscript and collecting clinical and other data.

**HV **participated in study design and coordination, critical revision of the manuscript and helped collecting clinical data.

**WH **contributed to the conception and design of the study, constructed the Markov model, did all Excel analyses and helped drafting the manuscript.

All authors have read and approved the final manuscript.

## Appendix

Word explanation: bi-allelic = two (i.e. homozygous or compound heterozygous) MUTYH mutations; a heterozygote/heterozygous mutation = one MUTYH mutation; non-bi-alellic = no (a non-carrier) or one MUTYH mutation (a heterozygote); proband or indexpatient = the affected subject who led to the research done on their family

## Pre-publication history

The pre-publication history for this paper can be accessed here:


